# Acculturation and health seeking behaviors in migrant adolescents: sample of Syrians living in Turkiye

**DOI:** 10.3389/fpubh.2026.1807338

**Published:** 2026-07-13

**Authors:** Zulal Soylu Karaca, Hande Yagcan, Dilek Bilgic

**Affiliations:** 1 Department of Midwifery, Faculty of Health Sciences, Istanbul Topkapi University, Istanbul, Türkiye; 2 Institute of Health Sciences, Dokuz Eylul University, Izmir, Türkiye; 3 Faculty of Nursing, Dokuz Eylul University, Izmir, Türkiye

**Keywords:** acculturation, adolescent health, health promotion, health seeking, migration

## Abstract

**Background:**

Health-seeking behaviors directly affect migrant adolescents' access to healthcare, health outcomes, and social integration. The aim of this study is determine the effect of acculturation on health-seeking behaviors in adolescent migrants aged 16–18 years.

**Methods:**

This descriptive and correlational cross-sectional study was conducted with 272 migrant adolescents, selected using convenience sampling, who completed the Individual Identity Form, the Health-Seeking Behavior Scale (HSBS) and the Vancouver Index of Acculturation (VIA). Data were collected between April 2023-April 2024 and analyzed using IBM SPSS Statistics 27.

**Results:**

Traditional health-seeking behavior and VIA core culture orientation had higher mean scores. mainstream culture orientation had positive and significant effect on health seeking behavior (*p* = 0.044).

**Conclusion:**

Educated, socially secure, living mostly in cities with their nuclear families, and with income equal to expenditure, adolescent migrants scored significantly higher on the health-seeking behavior scale. These relationships revealed that cultural orientations have a significant effect on health seeking behaviors. Future studies with larger, diverse samples are recommended.

## Introduction

The concept of illness, like health, is a cultural phenomenon that varies from society to society and over time, and therefore cannot be considered solely as a biological process. A condition considered an illness in one society may be considered normal in another. Culture influences individuals' perceptions of health, illness, and death, their beliefs about the causes of illness, their approaches to health promotion, their help-seeking behaviors, and their preferred treatment methods; while personal hygiene, economic status, family structure, gender roles, marriage and sexual behaviors, attitudes toward birth control, dietary habits, and immigration status are among the key cultural factors determining health-seeking behaviors (1).

While understanding the health policy systems of countries can be challenging even for locals, this situation becomes almost impossible for immigrants. Financial barriers, language barrier and attitudes of health professionals make this situation even more difficult. During adolescence, when identity development, independent decision-making abilities and susceptibility to risky behaviors are already critical, migrant adolescents face additional barriers such as language differences, cultural conflicts, unfamiliarity with the healthcare system and stigmatization. These can negatively affect their health-seeking behaviors (2–4).

As a result of the rapid increase in the number of migrant refugees, especially in European countries, understanding how to access and follow up the health services they need is the first step to overcome the difficulties they may face (5–7). Factors affecting the health-seeking behaviors of migrants in Europe include medical science, health communication and many disciplines. In the studies, the importance of the roles played by religion, belief and cultural norms in the health service seeking behaviors of migrants is emphasized. Studies reveal the importance of adopting an intercultural approach especially in the provision of health services. The experiences of forced migrants such as refugees such as war, stigmatization, deprivation, marginalization before acculturation make acculturation processes difficult (8, 9) examined the acculturation levels of international students in their study and found that those who migrated voluntarily and for a purpose adapted more easily to the country they came from. Migration is a phenomenon that places considerable strain on individuals, while affecting certain groups more severely than others. One such group is adolescents. Adolescents who are forced to migrate may encounter multiple stress factors related to relocation, acculturation, and adaptation to new educational environments. Shatanawi's study highlights that social isolation and loneliness are seen as significant psychiatric difficulties among Syrian refugee adolescents, particularly among those who have been unable to adapt culturally (10–12).

WHO defines adolescents as the 10–19 age group (13). When the literature is examined, studies investigating the effect of acculturation on health-seeking behaviors in migrant individuals are limited. For all these reasons, our study aimed to reveal the effect of acculturation on health-seeking behaviors in adolescent migrants aged 16–18 years.

## Materials and methods

### Study design

This descriptive and correlational cross-sectional study was reported in accordance with the STROBE guidelines for observational studies and it was conducted with a convenience sample of 272 adolescent migrants aged 16–18 years who applied to the outpatient clinics of S.B.Ü. Dr. Behçet Uz Children's Diseases and Surgery Training and Research Hospital in Izmir, the third largest city in Türkiye. The sample size (*n* = 272) was determined based on the known population of adolescent migrants (aged 16–18) who applied to the hospital within the past year. In the study, the relationship between acculturation status and health seeking of adolescent migrants was evaluated. People who did not want to participate in the study, who had a physical disability that would prevent them from moving (such as vision, mobility), who were diagnosed with a psychiatric illness and who were over 18 years old were excluded from the study. Approval for the research was received from the XXX University Non-Interventional Research Ethics Committee (no:2022/27-21). Data were collected between April 2023-April 2024 and analyzed using IBM SPSS Statistics 27.

The power of the study was calculated with G-Power program and *post-hoc* power analysis was used. The convenience sampling method was used. When the relationship between the Health Seeking Behaviors Scale and the Mainstream Acculturation Subscale was taken as 0.156, 82% power was reached with a 5% margin of error and a sample size of 272. Since the power of the research is above 80%, it is within the acceptable limit. Statistical significance was accepted as *p* < 0.05.

### Data collection

Demographic data were collected with the Individual Identification Form created by the researchers in line with the literature. The individual introduction form developed by the researchers in line with the literature consists of 13 questions related to the socio-demographic characteristics of adolescent migrants (9, 10). Health-seeking behavior and cultural adaptation were assessed using the Health-Seeking Behaviors Scale (14) and the Vancouver Index of Acculturation (VIA) (15) through face-to-face interviews lasting approximately 15 min. The Health-Seeking Behaviors Scale was translated into Arabic with the assistance of an interpreter during the interviews, and the total Cronbach's alpha coefficient for the scale in this study was calculated as 0.784. A validated and reliable version of the Vancouver Index of Acculturation (VIA) exists in Arabic, and this study used the Arabic form of the scale.

The Health-Seeking Behaviors Scale, developed by Kiraç and Öztürk, consists of 12 items and three subdimensions: online, professional, and traditional health-seeking behaviors. Items are rated on a 5-point Likert scale, with higher scores indicating more positive health-seeking behaviors. The Cronbach's alpha coefficient of the scale was reported as 0.755 in the original study, and it was found to be 0.784 in the present study, indicating acceptable reliability.

The Vancouver Index of Acculturation (VIA), developed by Ryder et al. and adapted into Turkish and Arabic by Bozdag and Bilge, was used to assess cultural orientation. The scale consists of 20 items and two subdimensions: core culture orientation and mainstream culture orientation, each including 10 items. Items are rated on a 9-point Likert scale, with higher scores indicating stronger cultural orientation. Acculturation strategies (integration, assimilation, separation, marginalization) were determined based on the mean scores of the two subdimensions. In the present study, Cronbach's alpha coefficients were 0.86 for core culture orientation and 0.84 for mainstream culture orientation.

### Analyzing the data

First, the Kolmogorov-Smirnov test was used in the normality analysis of the data, and due to the normal distribution of the data, the T-test from parametric tests and One Way ANOVA test were used to calculate the significance of the difference between independent means. Number, percentage and frequency analyses of socio demographic data were performed. The mean scores of the acculturation index and health seeking behaviors scale of adolescent migrants were compared with the significance test of the difference between two means from parametric tests and Pearson Correlation Analysis was performed. Statistical significance was accepted as *p* < 0.05. Pearson correlation analysis and multiple regression analysis were performed to examine the relationships between variables.

## Results

The Kolmogorov-Smirnov test indicated that the data were normally distributed (*p* > 0.05). Therefore, parametric tests were used. These findings show that adolescents with higher levels of education, having social security, and living in city center tend to have more positive health-seeking behaviors.

Of the migrant adolescents who participated in the study, 63.2% were female. 51.1% of the participants were 17 years old, 67.6% were primary school graduates, 72.1% were employed and 37.9% were students. The majority of the participants (73.2%) had less financial income than expenses, 90.4% were single, 54.4% lived in a nuclear family, 58.1% had an illiterate mother, 54.8% had a primary school graduate father, 61.4% had 4–6 siblings. While 43% of the participants spent their longest lifetime in the district, this rate was 42.6% in the city center, 68.8% had no social security, 56.6% had between 1 and 4 hospital admissions in the last year. 91.2% of the participants reported that they did not have any chronic disease, 90.4% reported that they did not have any medication that they used continuously, and 64.7% reported that there was no individual with chronic disease in the family. When the participants were asked about the obstacles they encountered in the health institution, the majority (47.1%) stated that the biggest difficulty was the language barrier/lack of interpreter.

Traditional health seeking behavior has the highest mean score of 4.22 points in health seeking behaviors of migrant adolescents. In the Vancouver Index of Acculturation (VIA), mean score of core culture orientation (63.00) is higher than the mean score of Mainstream Culture Orientation (52.68) (Table 1).

**Table 1:** Mean scores of the health seeking behaviors scale and vancouver acculturation index of immigrant adolescents.

Variable	Article number	Mean ±SD	Min	Max	Cronbach's alpha
Health seeking behaviors scale					
Online health search	6	2.58 ± 0.92	1	5	0.895
Professional health search	3	3.75 ± 0.68	2	5	0.762
Traditional health search	3	4.22 ± 0.69	2	5	0.845
Total	12	3.52 ± 0.47	2	5	0.784
Vancouver acculturation index					
Core culture orientation	10	63.00 ± 13.82	10	90	0.858
Mainstream culture orientation	10	52.68 ± 13.07	10	90	0.843

The relationships between health-seeking behaviors and cultural orientations of immigrant adolescents were examined using Pearson correlation analysis. The total score for health-seeking behaviors was found to be positively correlated with both online and traditional health-seeking behaviors, and to have a weak positive correlation with mainstream cultural orientation; conversely, traditional health-seeking behaviors were negatively correlated with mainstream cultural orientation, and core-cultural orientation was also negatively correlated with mainstream cultural orientation (Table 2).

**Table 2:** The relationship between the health seeking behaviors scale and vancouver acculturation index scores of migrant adolescents.

Variable	Category	Professional health search	Traditional health search	Health seeking behaviors scale	Core culture orientation	Mainstream culture orientation
Online health search	*r*	0.120^*^	−0.058	0.675^**^	−0.072	0.267
Professional health search	*r*		0.157^*^	0.636^**^	−0.055	0.138
Traditional health search	*r*			0.527^**^	0.098	−0.169
*Health seeking behaviors scale*	r				−0.025	0.156
Core culture orientation	*r*					−0.392

*r: Pearson Correlation Analysis*. ^*^p < 0.05, ^**^p < 0.01 values represent Pearson correlation coefficients.

Health Seeking Behavior Scale scores of immigrant adolescents showed significant differences according to educational status, socioeconomic characteristics, and living conditions. Adolescents who were illiterate scored lower on the scale compared to those who had completed primary or secondary school (*p* < 0.001), while adolescents who were students, had nuclear families, and had social security scored higher (*p* < 0.001). Those whose income was less than their expenses had lower health seeking behavior (*p* = 0.028), and adolescents whose parents had low educational levels and who spent most of their lives in villages also had significantly lower health seeking behavior scores (*p* < 0.001; *p* = 0.001) (Table 3).

**Table 3:** Comparison of the descriptive characteristics of migrant adolescents and the mean score of the health seeking behaviors scale.

Variable	Category	*n*	Mean ±SD	Statistics	*p*
Gender	Woman	172	3.52 ± 0.47	*t =* 0.083	0.934
	Male	100	3.51 ± 0.49		
Age	16	112	3.54 ± 0.49	*F* = 0.213	0.808
	17	139	3.50 ± 0.47		
	18	21	3.53 ± 0.46		
Education status	Illiterate^a^	84	3.28 ± 0.40	*F* = 18.170	**<0.001** **a** **<** **b, c**
	Primary education^b^	184	3.62 ± 0.46		
	High School^c^	4	3.96 ± 0.65		
	***Post-hoc*** **(Tukey)**				
Employment status	Yes	76	3.49 ± 0.46	*t =* −0.666	0.506
	No	196	3.53 ± 0.48		
Being a student	Yes	103	3.68 ± 0.49	*t =* 4.553	**<0.001**
	No	169	3.42 ± 0.44		
Income status	Income less than expenditure^a^	199	3.48 ± 0.47	*F* = 3.611	**0.028** **a** **<** **c**
	Income equals expenditure^b^	65	3.59 ± 0.43		
	Income more than expenditure^c^	8	3.86 ± 0.74		
	***Post-hoc*** **(Tukey)**				
Marital status	Married	26	3.45 ± 0.43	*t =* −0.731	0.465
	Single	246	3.52 ± 0.48		
Family structure	Nuclear family	148	3.61 ± 0.48	*t =* 3.490	**<0.001**
	Extended family	124	3.41 ± 0.45		
Mother's education level	Illiterate^a^	158	3.38 ± 0.41	*F* = 20.589	**<0.001** **a, b** **<** **c, d**
	Primary education^b^	87	3.60 ± 0.41		
	High School^c^	19	4.09 ± 0.56		
	University^d^	8	3.97 ± 0.56		
	***Post-hoc*** **(Tukey)**				
Father's education level	Illiterate^a^	67	3.41 ± 0.43	*F* = 13.096	**<0.001** **a, b** **<** **c, d**
	Primary education^b^	149	3.45 ± 0.42		
	High School^c^	45	3.74 ± 0.52		
	University^d^	11	4.13 ± 0.52		
	***Post-hoc*** **(Tukey)**				
Number of siblings	1–3 siblings	67	3.56 ± 0.54	*F* = 1.136	0.323
	4–6 siblings	167	3.52 ± 0.48		
	7 or more siblings	38	3.42 ± 0.28		
Place lived the longest	Province^a^	116	3.60 ± 0.46	*F* = 7.576	**0.001** **c <** **a, b**
	District^b^	117	3.51 ± 0.47		
	Village^c^	39	3.27 ± 0.43		
	***Post-hoc*** **(Tukey)**				
Social security status	Yes	85	3.69 ± 0.56	*t =* 4.297	**<0.001**
	No	187	3.44 ± 0.41		
Number of hospital admissions in the last year	1 to 4	154	3.55 ± 0.47	*t =* 1.285	0.200
	5 and above	118	3.48 ± 0.47		
Presence of chronic disease	Yes	24	3.52 ± 0.41	*t* = 0.010	0.992
	No	248	3.52 ± 0.48		
Presence of continuous medication	Yes	26	3.38 ± 0.36	*t* = −1.584	0.114
	No	246	3.53 ± 0.48		
Presence of chronic disease in the family	Yes	96	3.47 ± 0.47	*t* = −1.318	0.189
	No	176	3.55 ± 0.47		
The obstacle encountered in the health institution	Language barrier/lack of interpreter	128	3.48 ± 0.48	*F* = 0.639	0.590
	Financial difficulties/inability to buy medication	49	3.51 ± 0.45		
	Inability to find an appointment	61	3.57 ± 0.46		
	Negative behavior of health personnel	34	3.57 ± 0.48		

*t*: Independent Sample *t* Test; *F*: One Way ANOVA. *Post hoc* comparisons were performed using Tukey's test. Bold values indicate statistically significant results (*p* < 0.05). Different superscript letters indicate significant differences (Tukey post hoc, *p* < 0.05).

Adolescents with low educational levels and those who are married had higher scores on self-cultural orientation, while those who are students, single, from nuclear families, and with fewer than seven siblings had higher scores on widespread cultural orientation. Furthermore, adolescents experiencing obstacles due to negative attitudes from healthcare personnel had lower scores on widespread cultural orientation (Table 4).

**Table 4:** Comparison of the descriptive characteristics of immigrant adolescents and the mean scores of the vancouver acculturation index subscale.

		Core culture orientation		Mainstream culture orientation	
Variable	Category	Mean ±SD	Statistics	Mean ±SD	Statistics
Gender	Woman	61.91 ± 14.46	*t =* −1.702 *p* = 0.090	53.27 ± 13.95	*t =* 0.988 *p* = 0.090
	Male	64.86 ± 12.48		51.65 ± 11.40	
Age	16	64.40 ± 13.27	*F* = 1.113 *p* = 0.330	53.17 ± 12.83	*F* = 0.135 *p* = 0.874
	17	61.80 ± 13.97		52.32 ± 13.48	
	18	63.43 ± 15.58		52.38 ± 12.01	
Education status	Illiterate^a^	63.23 ± 15.67	*F* = 3.814 ***p*** **=** **0.023**	50.83 ± 14.30	*F* = 1.764 *p* = 0.173 **c** **<** **a, b**
	Primary education^b^	63.30 ± 12.60		53.35 ± 12.39	
	High School^c^	44.25 ± 17.17		60.25 ± 14.20	
	***Post-hoc*** **(Tukey)**				
Employment status	Yes	63.21 ± 14.14	*t =* 0.159 *p =* 0.874	51.04 ± 12.83	*t =* −1.288 *p =* 0.199
	No	62.91 ± 13.73		53.31 ± 13.14	
Being a student	Yes	62.58 ± 12.38	*t =* −0.385 *p =* 0.701	54.82 ± 12.64	*t =* 2.121 ***p** **=*** **0.035**
	No	63.25 ± 14.66		51.37 ± 13.19	
Income status	Income less than expenditure	62.44 ± 14.39	*F* = 2.644 *p =* 0.073	52.74 ± 13.37	*F* = 0.171 *p =* 0.843
	Income equals expenditure	65.65 ± 11.48		52.20 ± 12.11	
	Income more than expenditure	55.25 ± 13.69		55.00 ± 14.26	
Marital status	Married	68.19 ± 12.33	*t =* 2.028 ***p** **=*** **0.044**	44.38 ± 8.16	*t =* −3.471 ***p** **=*** **0.001**
	Single	62.45 ± 13.88		53.55 ± 13.19	
Family structure	Nuclear family	62.73 ± 13.17	*t =* −0.347 *p =* 0.729	54.34 ± 12.64	*t =* 2.309 ***p** **=*** **0.022**
	Extended family	63.31 ± 14.60		50.69 ± 13.34	
Mother's education level	Illiterate	62.50 ± 15.18	*F* = 0.381 *p =* 0.767	51.70 ± 13.83	*F* = 1.818 *p =* 0.144
	Primary education	63.91 ± 11.46		52.97 ± 11.83	
	High school	64.26 ± 10.35		56.00 ± 11.04	
	University	59.88 ± 17.41		61.00 ± 12.55	
Father's education level	Illiterate	62.06 ± 14.80	*F* = 0.526 *p =* 0.665	51.01 ± 13.38	*F* = 2.323 *p =* 0.075
	Primary education	63.26 ± 13.82		52.19 ± 13.20	
	High school	64.40 ± 11.29		54.73 ± 12.15	
	University	59.36 ± 17.63		61.00 ± 10.05	
Number of siblings	1–3 siblings^a^	63.75 ± 12.33	*F* = 2.740 *p =* 0.066	54.94 ± 12.63	*F* = 4.182 ***p** **=*** **0.016** **c** **<** **b** **<** **a**
	4–6 siblings^b^	61.71 ± 14.78		52.96 ± 13.08	
	7 or more siblings^c^	67.34 ± 10.94		47.45 ± 12.68	
	***Post-hoc*** **(Tukey)**				
The place where he lived the longest	Province	64.68 ± 13.75	*F* = 1.561 *p =* 0.212	53.53 ± 13.10	*F* = 0.431 *p =* 0.650
	District	61.95 ± 13.81		52.11 ± 13.78	
	Village	61.13 ± 13.85		51.85 ± 10.70	
Presence of social security	Yes	61.24 ± 16.54	*t =* −1.420 *p =* 0.157	53.67 ± 15.29	*t =* 0.845 *p =* 0.399
	No	63.80 ± 12.36		52.22 ± 11.94	
Number of hospital admissions in the last year	1 to 4	62.08 ± 14.20	*t =* −1.253 *p =* 0.211	52.56 ± 13.59	*t =* −0.161 *p =* 0.873
	5 and above	64.19 ± 13.27		52.82 ± 12.40	
Presence of chronic disease	Yes	65.08 ± 14.58	*t =* 0.774 *p =* 0.439	50.58 ± 11.13	*t =* −0.821 *p =* 0.412
		
	No	62.79 ± 13.76		52.88 ± 13.24	
Presence of continuous medication	Yes	64.73 ± 15.76	*t =* 0.672	50.62 ± 11.50	*t =* −0.845
	No	62.81 ± 13.62	*p =* 0.502	52.89 ± 13.22	*p =* 0.399
Presence of chronic disease in the family	Yes	60.66 ± 16.39	*t =* −2.075 ***p** **=*** **0.039**	54.00 ± 13.64	*t =* 1.235 *p =* 0.218
	No	64.27 ± 12.05		51.95 ± 12.73	
The obstacle encountered in the health institution	Language barrier/lack of interpreter^a^	63.53 ± 12.69	*F* = 2.003 *p =* 0.114	52.78 ± 12.65	*F* = 2.747 ***p** **=*** **0.043** **d** **<** **b**
	Financial difficulties/inability to buy medication^b^	59.43 ± 16.39		55.33 ± 13.73	
	Inability to find an appointment^c^	62.67 ± 13.11		53.36 ± 13.60	
	Negative behavior of health personnel^d^	66.71 ± 14.49		47.24 ± 11.57	
	***Post-hoc*** **(Tukey)**				

*t:* Independent Sample *t* Test; *F*: One Way ANOVA. *Post hoc* comparisons were performed using Tukey's test. Bold values indicate statistically significant results (*p* < 0.05). Different superscript letters indicate significant differences (Tukey post hoc, *p* < 0.05).

## Discussion

This study demonstrates that health-seeking behaviors in immigrant adolescents are significantly influenced by the cultural adaptation process, and that cultural orientations play a decisive role in health-seeking behaviors. Considering the limited number of studies in the literature focusing on health-seeking behaviors in immigrant groups, particularly adolescents, these findings are thought to make a significant contribution to this field. In Turkey, studies focusing on migrant adolescents are still limited and research specifically addressing health-seeking behaviors is particularly scarce. Existing studies mostly focus on health service utilization, but the differences between traditional and professional health-seeking behaviors are not adequately addressed. This indicates a significant gap, and this study aims to fill that gap in domestic and foreign literature. Our findings show that sociodemographic variables such as education level, family structure, economic status, health insurance coverage, and place of residence significantly influence health-seeking behaviors. The lower health-seeking behaviors of adolescents who are illiterate or have a low level of education are consistent with previous studies on immigrant groups (16, 17).

The highest average for traditional health-seeking behaviors in our study indicates that immigrant adolescents tend to gravitate toward traditional methods rather than professional healthcare services. In the current literature, this is explained by language barriers, difficulties in adapting to the healthcare system, economic constraints, limited health insurance coverage, uncertainties regarding the cost of healthcare services, and concerns related to legal status (18, 19). Furthermore, negative attitudes and perceptions of discrimination among healthcare professionals are also significant factors limiting the use of professional healthcare services. In this context, it can be said that the tendency toward traditional methods is a result not only of cultural preferences but also of structural and systemic barriers (20, 21).

In our study, adolescents with extended family structures and who are more attached to their core culture were found to have higher scores for traditional health-seeking behavior. This finding is consistent with studies showing that cultural attachment and behavioral continuity can limit healthcare use in non-Western immigrant groups (22). Similarly to literature, our findings show that immigrants without health insurance and living in rural areas have lower health-seeking behaviors indicate that unmet healthcare needs are common in immigrant groups (23).

The Vancouver Index of Acculturation results show that participants' self-cultural orientations are higher than their mainstream cultural orientations. However, adolescents with higher mainstream cultural orientations were also found to have more positive health-seeking behaviors. This indicates that cultural adaptation facilitates access to healthcare services and strengthens the behavior of seeking professional healthcare. Specifically, our study found that language barriers constitute a stronger obstacle than material difficulties and have a significant negative impact on mainstream cultural orientation. Current literature emphasizes that knowing the host country's language is one of the key determinants of cultural adaptation and that language proficiency increases health service utilization (18, 19, 21).

The acculturation process can be considered not only as a process of social adaptation but also as one that indirectly affects the psychosocial well-being of adolescent immigrants. Factors such as social exclusion, language barriers, and discrimination create stress and difficulties in adaptation; conversely, social support and integration support more positive coping processes (24, 25). In her study examining immigrants aged 13–17, Tiryaki (24) revealed that participants largely exhibited an integration-oriented acculturation process, but also showed tendencies toward separation depending on factors affecting the adaptation process. Among these factors, length of residence in the country, early arrival, and the presence of social support positively influenced acculturation, while language barriers and socioeconomic difficulties stood out as significant hindering factors (24). In the qualitative research conducted by Agcadar Çelik and Çetin with Syrian adolescents in Hatay, similar to our findings, it was observed that immigrant adolescents tend toward integration and acculturation processes (25).

Although traditional health-seeking behaviors were the highest sub-dimension, the above-average scores for online and professional health-seeking behaviors suggest that the immigrant adolescents participating in the study show a tendency toward integration in terms of cultural adaptation. While studies directly examining the relationship between cultural adaptation levels (assimilation, integration, separation, marginalization) and health service utilization are limited in the literature, it is reported that increased adaptation to the mainstream culture positively affects health service utilization and health-seeking behaviors (26, 27).

When we look at examples from Turkey, the primary problem faced by immigrants in accessing healthcare services is the language barrier. It has been emphasized that to overcome this problem, Turkish language training should be provided or qualified translators should be employed. In addition, financial barriers, transportation difficulties, and differences in immigrant status are also cited as significant factors hindering access to healthcare services (28).

## Limitation

The strength of our study is that it is the first study to evaluate all dimensions of the acculturation effect on health seeking behaviors in immigrant individuals. However, the current study has some limitations. For the adaptation of the Vancouver Index of Acculturation, since the study was conducted with Syrian migrant groups aged 11–18, the late adolescent group (16–18 years) was selected in line with the recommendations of the index author.

Migrant adolescent patients consisted of only one children's hospital and the city. It cannot be generalized to other populations. Another limitation is that the scales are self-report.

## Conclusion

This study, the first to examine the relationship between acculturation and health-seeking behaviors among Syrian adolescent migrants in Turkey, found that although the core-culture sub-orientation was higher than the mainstream culture sub-orientation, both orientations were above the average value and the participants were found to be in the integration sub-category. If we look at the markers of health-seeking behavior it was observed that immigrant adolescents, especially those with extended family structure, concentrated on traditional health-seeking behaviors. Among the similar factors affecting health-seeking behaviors and acculturation levels of individuals, language barrier, parents' education level and personal education were found to be important factors. It is seen that adolescents who are oriented to the mainstream culture show more interest in online health and general health seeking behaviors, while adolescents who are oriented to the core culture show more tendency toward traditional health seeking behaviors. These relationships revealed that cultural orientations have a significant effect on health seeking behaviors. It is seen that the adaptation of immigrants to the cultures of the countries they migrate to will positively affect their health status. In future studies, it is recommended to examine the effect of these interventions on health-seeking behaviors by determining and supporting the adaptation of immigrants to the culture of the country of immigration with a larger sample including all immigrant age groups.

This study provides time- and socially significant descriptive evidence regarding the relationship between acculturation and health-seeking behaviors in Syrian migrant adolescents living in Turkey. The dominance of traditional health-seeking behaviors suggests that interventions should move beyond individual-centered approaches and target trusted figures within the family and community. The weak positive correlation between dominant cultural orientation and total health-seeking behavior, and the negative correlation with traditional seeking behavior, indicate that adolescents diversify their health-related resources during the acculturation process. In this context, considering the prevalence of language barriers, investments in interpreters, cultural mediators, and adolescent-friendly referral services can be said to have high potential impact.

## Data Availability

The raw data supporting the conclusions of this article will be made available by the authors, without undue reservation.
